# A dataset of paired blood mRNA and microRNA sequencing across acute septic shock and recovery

**DOI:** 10.1038/s41597-026-06844-w

**Published:** 2026-02-14

**Authors:** Krisztina Molnár, Katalin Maricza, Zsuzsanna Elek, Réka Kovács-Nagy, Ábel Fóthi, Zsófia Bánlaki, Eszter Losoncz, Bernadett Húri, János Kádas, Gergely Keszler, Zsolt Rónai

**Affiliations:** 1 Institute of Biochemistry and Molecular Biology, Department of Molecular Biology, Semmelweis University, Budapest, Hungary; 2 Doctoral School, Semmelweis University, Budapest, Hungary; 3 Klinik Oberwart, Abteilung für Anästhesiologie und Intensivmedizin, Oberwart, Austria; 4 Péterfy Sándor Street Hospital and Outpatient Clinic, Budapest, Hungary; 5 UD-GENOMED Medical Genomic Technologies Ltd., Debrecen, Hungary

**Keywords:** Prognostic markers, Infection

## Abstract

An adequate immune response is responsible for eradicating pathogens and reestablishing tissue homeostasis upon infection. However, in certain patients, immune processes become dysregulated, leading to sepsis which often results in life-threatening organ dysfunction, and the progression to septic shock is associated with mortality rates of up to 70–80%. The objective of the present data set is to facilitate the identification of transcriptomic signatures characteristic of septic shock and the stable, out of critical condition. A total of six patients were included in the study, with blood samples collected at two different stages – septic shock, at the time of discharge from the intensive care unit – of the disease. Following total RNA isolation, mRNA and microRNA levels were determined by NGS. The dataset’s significance is based on the fact that two samples of the same patient were analyzed, ensuring that any observed alteration in transcript levels is related to the change in medical condition, and the analysis included both mRNAs and microRNAs enabling a comprehensive gene expression and regulatory study.

## Background & Summary

The dynamics of cellular and humoral immune responses to bacterial, viral and fungal infections are governed by the coordinated action of pro- and anti-inflammatory cytokines. The resulting finely tuned immune response first elicits an acute inflammatory reaction aimed at eliminating pathogens. At a later stage, it helps restore homeostasis in damaged tissues. However, in some cases, an elusive interaction between pathogen virulence signatures and the host immune system can lead to sepsis, a dysregulated immune response to infection characterized by uncoordinated production of cytokine mediators and life-threatening organ dysfunction^1^. The clinical manifestation of sepsis changes continuously and the condition of affected patients may deteriorate unexpectedly despite administration of targeted antimicrobial agents. Therefore, setting up an early diagnosis and initiating adequate organ support therapy is paramount to improve the chances of patient recovery^2^. Despite significant progress in our understanding of the pathophysiology of sepsis and the expansion of therapeutic interventions in recent years, sepsis continues to be the foremost cause of mortality^3,4^. Therefore, there is an urgent need for a reliable and expeditious diagnostic marker that can be effectively utilized in the clinical setting. These aims are particularly relevant in the case of sepsis associated with community-acquired pneumonia (CAP). In a prospective observational study conducted at Osaka University, integrative mRNA and microRNA transcriptomic analyses were performed in 14 critically ill patients with CAP-associated sepsis compared to 15 healthy control subjects. All patients met the Sepsis-3 criteria, and six of them subsequently developed septic shock. RNA sequencing revealed significant differential expression levels with 1,209 mRNAs exhibiting increased expression while 1,461 were downregulated. Additionally, 51 microRNAs were found significantly upregulated, while transcript levels of 21 mRNAs were reduced. Furthermore, 646 microRNA-targeted mRNAs were overexpressed as compared to 1,274 with reduced expression (all at FDR < 0.05, |log_2_FC| > 1.2). The pathway analysis indicated activation of PD-1/PD-L1 immune checkpoint signaling and inhibition of Th1 immune responses. This integrative analysis was the first to demonstrate T-cell exhaustion and microRNA-mediated modulation of critical immune pathways in the acute phase of CAP-related sepsis^5^, https://www.ncbi.nlm.nih.gov/geo/query/acc.cgi?acc = GSE228541; https://www.ncbi.nlm.nih.gov/geo/query/acc.cgi?acc = GSE228540.

Complementing these mechanistic findings, a multi-center UK-based study focused on the diagnostic differentiation of sepsis and systemic inflammatory response syndrome (SIRS) through whole-blood mRNA expression profiling. The study included 59 patients with abdominal and a further 84 with pulmonary sepsis, and 42 with SIRS due to out-of-hospital cardiac arrest, along with 30 healthy controls. Samples were taken at four clinical time points during treatment in the intensive care unit. A panel of 19 high-performance “indicators of inflammation” – including CD177, FAM20A, and OLAH – distinguished systemic inflammation from health (FC > 20, p < 0.05). Additionally, 20 “SIRS or Sepsis” biomarkers, such as CMTM5, CETP, PLA2G7, MIA, and MPP3, differentiated sepsis from SIRS with high accuracy (AUCROC = 0.9758). While validated in external datasets, some performance variability was noted, potentially due to differences in platforms and protocols^6^, https://www.ncbi.nlm.nih.gov/geo/query/acc.cgi?acc = GSE236713.

A separate investigation explored the potential of preoperative gene expression analysis to predict the likelihood of postoperative infection or sepsis. This investigation was part of a broader effort to expand the translational application of transcriptomics in medical research. Employing machine learning models that were trained on transcriptomic signatures from patients undergoing major elective surgery, individuals were successfully stratified by risk for postoperative sepsis. The models were further validated using RNA sequencing from patients with confirmed cases of moderate to severe pneumonia due to coronavirus infection^7^, https://www.ncbi.nlm.nih.gov/geo/query/acc.cgi?acc = GSE208587.

These data demonstrate that transcriptomic profiling, comprising both mRNA and microRNA, has emerged as a potent approach to elucidate the molecular mechanisms underlying immune dysregulation. This approach can contribute to the identification of diagnostic biomarkers and to the prediction of individual susceptibility to sepsis. As indicated by the extant literature, interindividual differences in host genetics have been demonstrated to influence sepsis risk and outcomes by modulating various immune processes, including but not limited to pathogen recognition, cytokine secretion, and intracellular signaling^8^.

Rautanen *et al*. conducted a search for single nucleotide variations (SNVs) that influence the 28-day survival of patients with sepsis^9^. Despite the fact that the researchers described a number of highly significant associations between sepsis and genetic variations in a cluster of candidate genes, including the FER non-receptor tyrosine kinase, none of their findings could be replicated by another methodologically and statistically robust GWAS by Scherag and colleagues^10^. This study identified a missense marker SNV in the VPS13A gene and a non-coding variant in the CRISPLD2 gene.

The data set presented here is of particular scientific value for two reasons. The analysis encompassed two samples from each patient, with one sample corresponding to severe, life-threatening septic shock according to Sepsis-3 guidelines, while the other representing a clinically stable (out of critical) state following resolution of a dysregulated immune response. Comparing these corresponding sample sets is particularly informative because, given that they originate from the same individual, the transcriptomic differences observed are more likely to be related to changes in the clinical condition rather than comparing cohorts of different individuals. Another strength is that all samples were analyzed for both mRNA and microRNA, enabling a more intricate analysis of gene expression. This provides information not only on transcriptional activity but also gives an insight into post-transcriptional regulatory circuitries.

The major limitation of the dataset is its relatively limited sample size. Therefore, analysis of these transcriptomic data can only facilitate exploration of trends and generation of hypotheses. However, it is evident that results obtained from a higher number of patients would be necessary for robust biomarker discovery or definitive conclusions about disease pathogenesis. It is also important to note that the origin of sepsis was not the same in all cases examined: three patients suffered from respiratory tract infections and three from urogenital infections. The sample examined is, therefore, less homogeneous, but the different starting points may offer an opportunity to obtain initial information about transcriptomic patterns associated with the development of unregulated immune response and septic shock as the “final outcome”, regardless of the origin of the disease.

## Methods

### Patients, sample collection and RNA extraction

Six patients participated in the study, who were admitted to the Department of Anaesthesiology and Intensive Therapy of the Péterfy Sándor Street Hospital and Outpatient Clinic in Budapest. Patients were informed about the ongoing research at the time of hospital admission and/or during treatment, and they joined the study on a voluntary basis. Sampling was performed after obtaining written informed consent. The participants consented to transcriptomic analyses and to the publication of the resulting data on the condition that personal data would not be disclosed in any form. Protection of the identities of participants was ensured by assigning a unique code to each sample after collection. Ethics approval was granted by the National Centre for Public Health and Pharmacy, Department of Clinical Research (permission No.: NNGYK/GYSZ/10177-5/2024). This study was conducted in accordance with the WMA Declaration of Helsinki, and the handling of patient data was in compliance with the General Data Protection Regulation (GDPR) of the European Union. The participants were Hungarian citizens of Caucasian origin.

All patients had an underlying infectious disease confirmed by physical examination, laboratory tests and microbiological findings. The origin of sepsis was respiratory infection in three cases, whereas in the other half of samples urogenital infection.

Two samples were collected from each participant. The first sample corresponded to the septic shock state based on the Third International Consensus Definitions for Sepsis and Septic Shock^1^, while the second one was taken upon discharge from ICU, representing the post-critical stable state (Table 1). The improvement of clinical status was underpinned by clinical parameters including SOFA assessment scores and vasopressor therapy (Table 2). Although there was no statistically significant difference in procalcitonin (PCT) levels between the two clinical stages in the entire cohort, this can be explained by the fact that PCT concentrations are known to vary greatly from person to person. Comparison of individual PCT levels in sepsis vs. stable (out-of-critical condition) sample pairs (B1: 5.42 → 0.4; B2: 6.32 → 0.24; B3: 0.19 → 0.09; B4: 42.0 → 0.38; B5: 136.0 → 46.5; B6: 19.1 → 0.98) clearly demonstrated improvements in the clinical status. Table 3 summarizes further phenotypic and clinical data for each patient.

**Table 1:** Parameters of the RNA sample pairs obtained from the patients in septic shock and stable (out of critical) condition, respectively.

Patient, origin of sepsis	Clinical condition	RNA sample ID	RNA concentration (ng/µL)	RIN
B1, respiratory	septic shock	SZ372	232.03	8.5
	stable	SZ374	46.24	8.1
B2, urogenital	septic shock	SZ335	37.7	7.9
	stable	SZ340	39.113	7.8
B3, respiratory	septic shock	SZ341	58.249	7.5
	stable	SZ347	68.2	7.9
B4, urogenital	septic shock	SZ350	35.834	7.4
	stable	SZ352	61.39	7.9
B5, urogenital	septic shock	SZ356	919.01	8.4
	stable	SZ357	45.324	7.8
B6, respiratory	septic shock	SZ329	41.764	7.8
	stable	SZ334	33.106	7.2

RIN: RNA integrity number.

**Table 2:** Clinical parameters (average ± standard deviation) of the investigated patients in septic shock and stable recovery, respectively.

Parameter	Septic shock	Stable	Wilcoxon p
SOFA	9.00 ± 0.65	3.33 ± 0.55	0.0313
Noradrenaline treatment (µg/kg/min)	0.20 ± 0.04	not required	n/a
PCT (µg/L)	34.84 ± 23.64	8.1 ± 8.59	0.1250

Statistical levels (*p*) of difference between the two conditions was assessed by Wilcoxon Matched Pairs Test. PCT: Procalcitonin.

**Table 3:** Detailed phenotype data of the patients.

Patient	Age	Gender	BMI	SOFA	Origin	Pathogen	NA treatment	PCT (µg/L)	CRP (mg/L)	FiO_*2*_	WBC (G/L)	THR (G/L)	creatinine (µmol/L)
B1 shock	47	male	31.8	10	respiratory	*E. coli*	0.2	5.42	339.3	0.6	13.2	145	86
B1 stable				5			not required	0.40	107.8	0.3	18.4	326	24
B2 shock	83	female	31.3	11	urogenital	*Pseudomonas aeruginosa*	0.125	6.32	118.9	0.3	26.2	97	177
B2 stable				3			not required	0.24	61.7	0.3	5.2	53	71
B3 shock	52	male	29.1	7	respiratory	*Klebsiella pneumoniae*	0.35	0.19	158.4	0.7	15.9	217	91
B3 stable				2			not required	0.09	36.2	0.21	12.3	349	55
B4 shock	72	female	34.0	8	urogenital	*E. coli*	0.2	42.00	343.1	0.9	19.1	110	133
B4 stable				4			not required	0.38	40.8	0.3	12.2	337	54
B5 shock	64	female	35.9	9	urogenital	*E. coli*	0.18	136.00	271.6	0.3	39.3	55	153
B5 stable				4			not required	46.50	180.7	0.3	14.4	79	77
B6 shock	46	female	26.6	9	respiratory	*Klebsiella pneumoniae*	0.14	19.10	123.1	0.8	1.9	97	61
B6 stable				2			not required	0.98	107.4	0.3	12.1	456	38
*Average shock*			*31.45*	*9.00*			*0.20*	*34.84*	*225.73*	*0.60*	*19.27*	*120.17*	*116.83*
*Average stable*				*3.33*				*8.10*	*89.10*	*0.29*	*12.43*	*266.67*	*53.17*

Age: Age at hospitalization, BMI: Body mass index (kg/m^2^), NA treatment: noradrenaline treatment (µg/kg/min), PCT: procalcitonin, FiO_2_: fraction of inspired oxygen, WBC: white blood cell count, THR: thrombocyte count.

Peripheral blood samples were collected into Tempus™ Blood RNA Tubes (Thermo Fisher). According to the manufacturer’s protocol, the collecting tubes were stored at room temperature for no longer than 5 days and then at –20 °C until further processing. Total RNA was isolated using the MagMax™ for Stabilized Blood Tubes RNA Isolation Kit. The average RNA concentration of the samples was 134.83 ng/µL (range: 33.106–919.01 ng/µL).

### mRNA and miRNA sequencing library preparation

The preparation of RNA-Seq libraries from total RNA was conducted using the Ultra II RNA Sample Prep kit (New England BioLabs, Inc., Ipswich, MA, USA), following the manufacturer’s protocol. In summary, poly-A RNAs were captured by means of oligo-dT-conjugated magnetic beads, thereafter, the mRNAs were eluted and fragmented at 94 °C. The first strand of cDNA was generated by random priming reverse transcription, which was followed by the synthesis of the second strand of the cDNA. After repairing cDNA ends, A-tailing, and adapter ligation steps, the adapter-ligated fragments were amplified in enrichment PCR. Subsequently, sequencing libraries were generated.

The NEXTFLEX Small RNA-Seq Kit v4 with UDIs (Revvity, Waltham, MA, USA) was utilized to generate small RNA sequencing libraries, in accordance with the manufacturer’s protocol. In summary, a total of 1 µg of RNA per sample was used for library preparation. The initial step was the ligation of the adenylated oligonucleotide adapter to the 3′ end. This was followed by the immediate execution of the 5′ adapter ligation. Following the completion of the two ligation steps, reverse transcription was initiated, resulting in the generation of first-strand cDNA. This first-strand cDNA was then purified through a process deploying magnetic beads. Subsequently, a PCR was performed, employing a NEXTFLEX UDI barcoded primer mix. Each sample was amplified by means of a distinct barcoded primer. In the final step of the procedure, the amplified products were purified and size-selected using AMPure magnetic beads.

### mRNA and miRNA sequencing

Normalized, pooled, and denatured library samples were subjected to sequencing on the Illumina NextSeq platform (Illumina, Inc., San Diego, CA, United States) using 100 bp SR chemistry with the NextSeq. 2000 P3 Kit (100 Cycles) to obtain global transcriptome data. The high-throughput mRNA and microRNA sequencing analysis yielded an average of 15 million and 10 million raw reads, respectively.

### Processing of sequencing data

Primer mRNA sequencing data (reads) were exported in fastq file format. The quality of original and trimmed reads was evaluated using FASTQC 0.12.1 (Babraham Bioinformatics). The reads were trimmed using Trim Galore 0.6.5 (Babraham Bioinformatics) and Cutadapt 5.1 software (Martin, M., 2011). Potential 3′-adapter and poly(A)-tail fragments were also removed from short reads using Cutadapt, with the 3′-adapter sequence specified and a stretch of 5′ adenines, respectively.

The raw miRNA sequencing data were then subjected to analysis using the StrandNGS 3.4 software. The fastq files were imported, and the small RNA aligner algorithm was used to align the raw reads to the human GRCH38 reference genome version 39. The small RNA gene models were inferred from the miRBase Sequence Database (Version 22.1) and Ensembl (Version 96) gene models. The alignment percentage was determined to be greater than 93%. The StrandNGS software’s integrated DESeq 2 algorithm was employed to quantify and normalize the aligned reads.

### Genome sequence and gene annotations, transcriptome sequence and alignment of mRNA sequencing data

The human GRCh38 genome sequence, version 39, was obtained from the NCBI database (/data10/genomes/Homo_sapiens/NCBI/hg38/Hisat2Index/genome; /data10/genomes/Homo_sapiens/NCBI/hg38/GCF_000001405.39_GRCh38.p13_genomic.gtf). Chromosomal sequences and annotation files were downloaded in FASTA and GTF formats, respectively. HISAT2 2.2.1 was used for indexing the genome and transcriptome sequences in FASTA format, as well as for the alignment of the trimmed sequence sets in fastq format to genome sequence reads (minimum percentage of matches: 90%)^11^. Aligned reads were collected in BAM files and aligned according to genomic coordinates using the SAMtools 1.22 program package^12^ before further use. The Rsubread R package’s FeatureCounts function was used to count reads for each gene^13^. Gene-based estimation of count-based gene expression level was performed. The results were summarized in a table where each row represents a transcript, and the read numbers for each sample are shown.

## Data Records

The primary sequence fastq files of mRNA and miRNA sequencing, processed data files along with the clinical status of the patients are available at Gene Expression Omnibus (GEO) database (National Center for Biotechnology Information (NCBI), United States) under the accession number of GSE296830. Table 4 summarizes the GEO accession numbers and file names for each read sets^14^ together with the library they correspond to.

**Table 4:** GEO accession numbers and libraries of the read sets.

*Patient*	*Status*	*RNA*	*Accession #*	*Library*	*File name*
B1	septic shock	mRNA	GSM8978217	SZ372mes	SZ372mes.fastq.gz
B1	septic shock	miRNA	GSM8978218	SZ372mic	SZ372mic.fastq.gz
B1	stable	mRNA	GSM8978221	SZ374mes	SZ374mes.fastq.gz
B1	stable	miRNA	GSM8978222	SZ374mic	SZ374mic.fastq.gz
B2	septic shock	mRNA	GSM8978195	SZ335mes	SZ335mes.fastq.gz
B2	septic shock	miRNA	GSM8978196	SZ335mic	SZ335mic.fastq.gz
B2	stable	mRNA	GSM8978199	SZ340mes	SZ340mes.fastq.gz
B2	stable	miRNA	GSM8978200	SZ340mic	SZ340mic.fastq.gz
B3	septic shock	mRNA	GSM8978201	SZ341mes	SZ341mes.fastq.gz
B3	septic shock	miRNA	GSM8978202	SZ341mic	SZ341mic.fastq.gz
B3	stable	mRNA	GSM8978205	SZ347mes	SZ347mes.fastq.gz
B3	stable	miRNA	GSM8978206	SZ347mic	SZ347mic.fastq.gz
B4	septic shock	mRNA	GSM8978207	SZ350mes	SZ350mes.fastq.gz
B4	septic shock	miRNA	GSM8978208	SZ350mic	SZ350mic.fastq.gz
B4	stable	mRNA	GSM8978211	SZ352mes	SZ352mes.fastq.gz
B4	stable	miRNA	GSM8978212	SZ352mic	SZ352mic.fastq.gz
B5	septic shock	mRNA	GSM8978213	SZ356mes	SZ356mes.fastq.gz
B5	septic shock	miRNA	GSM8978214	SZ356mic	SZ356mic.fastq.gz
B5	stable	mRNA	GSM8978215	SZ357mes	SZ357mes.fastq.gz
B5	stable	miRNA	GSM8978216	SZ357mic	SZ357mic.fastq.gz
B6	septic shock	mRNA	GSM8978191	SZ329mes	SZ329mes.fastq.gz
B6	septic shock	miRNA	GSM8978192	SZ329mic	SZ329mic.fastq.gz
B6	stable	mRNA	GSM8978193	SZ334mes	SZ334mes.fastq.gz
B6	stable	miRNA	GSM8978194	SZ334mic	SZ334mic.fastq.gz

A tab-delimited text file containing the raw counts of mRNAs (messengerrna.txt) and miRNAs (microrna.txt) for each sample is also available.

## Technical Validation

RNA concentrations as well as the A260/280 and A260/230 optical density ratios were measured using a DeNovix spectrophotometer. The quality and integrity of the samples were assessed by determining the RNA integrity number (RIN) using a Bioanalyzer 2100 instrument (Agilent Technologies, Inc., Santa Clara, CA, USA) employing the Eukaryotic Total RNA Nano Kit (RNA 6000) (Agilent Technologies, Inc., Santa Clara, CA, USA) according to manufacturer’s protocol (Table 1).

The final sequencing libraries were analyzed using a Bioanalyzer 2100 instrument (Agilent Technologies, Inc., Santa Clara, CA, USA) with an Agilent DNA 1000 Kit for mRNA libraries and Agilent High Sensitivity DNA Kit for miRNA libraries according to the manufacturer’s protocol. This process was used to verify the size and validate the quality of the libraries. The obtained parameters confirmed that each library was suitable for subsequent processing (Tables 5, 6).

**Table 5:** Validation of the mRNA sequencing libraries.

*RNA*	*Library*	*Size (bp)*	*Concentration*	
			*(ng/µL)*	*(nmol/L)*
SZ329	RS5503	299	43.13	218.3
SZ334	RS5504	331	37.80	173.2
SZ335	RS5505	356	20.59	87.7
SZ340	RS5507	301	47.41	238.7
SZ341	RS5508	329	26.67	122.9
SZ347	RS5537	347	25.74	112.5
SZ350	RS5538	354	16.93	72.5
SZ352	RS5540	327	22.15	102.6
SZ356	RS5541	375	22.59	91.3
SZ357	RS5542	285	23.70	125.8
SZ372	RS5543	346	41.87	183.1
SZ374	RS5545	348	5.69	24.8

**Table 6:** Validation of the miRNA sequencing libraries.

*RNA*	*Library*	*Size (bp)*	*Concentration*	
			*(ng/µL)*	*(nmol/L)*
SZ329	miR0455	163	6.35	59.1
SZ334	miR0456	162	4.76	44.6
SZ335	miR0457	162	6.18	57.8
SZ340	miR0459	163	6.28	58.5
SZ341	miR0460	163	5.94	55.3
SZ347	miR0462	162	3.70	34.6
SZ350	miR0463	160	10.46	99.0
SZ352	miR4065	160	8.23	78.0
SZ356	miR0466	161	6.78	63.8
SZ357	miR0467	161	11.33	106.4
SZ372	miR0468	163	9.36	86.9
SZ374	miR0470	160	4.54	42.9

## Data Availability

No custom code has been used in this study for data analysis.

## Data availability

Data for this paper are publicly available on the GEO repository (https://www.ncbi.nlm.nih.gov/geo/query/acc.cgi?acc = GSE296830).
